# Efficacy of a Topical Esafoxolaner, Eprinomectin and Praziquantel Combination Against Most Commonly Found Metazoan Parasites of Client-Owned Cats in Greece

**DOI:** 10.3390/vetsci12040385

**Published:** 2025-04-19

**Authors:** Isaia Symeonidou, Georgios Sioutas, Athanasios I. Gelasakis, Frederic Beugnet, Elias Papadopoulos

**Affiliations:** 1 Laboratory of Parasitology and Parasitic Diseases, School of Veterinary Medicine, Faculty of Health Sciences, Aristotle University of Thessaloniki, 54124 Thessaloniki, Greece; gsioutas@vet.auth.gr (G.S.); eliaspap@vet.auth.gr (E.P.); 2 Laboratory of Farm Animal Anatomy and Physiology, Department of Animal Science and Aquaculture, School of Agricultural Production, Infrastructure and Environment, Agricultural University of Athens, 11855 Athens, Greece; gelasakis@aua.gr; 3 Boehringer Ingelheim Animal Health, 29 Av. Tony Garnier, 69007 Lyon, France; frederic.beugnet@boehringer-ingelheim.com

**Keywords:** feline parasitism, epizootiology, multiparasitism, Greece, NexGard^®^ Combo

## Abstract

Feline parasitism affects animals’ health and welfare. Faeces from 472 client-owned cats from Greece were examined to provide updated data on the epizootiology of metazoan endo- and ectoparasites (namely, *Toxocara cati*, Ancylostomatidae, *Dipylidium caninum*, lungworms, *Toxascaris leonina*, *Otodectes cynotis*, fleas, ticks and *Notoedres cati*). All positive animals received a topical formulation containing esafoxolaner, eprinomectin and praziquantel (NexGard^®^ Combo, Boehringer Ingelheim), and its efficacy was evaluated. The overall prevalence of parasitism was 22.9%, while that of multiparasitism was 16.3%. *Toxocara cati* (18.4%) was the most prevalent endoparasite, followed by Ancylostomatidae (10.8%), *D. caninum* (4.7%), lungworms (2.5%) and *T. leonina* (0.4%). Regarding ectoparasites, *O. cynotis* (3.2%), fleas (2.3%), ticks (0.6%) and *N. cati* (0.4%) were found. To estimate the efficacy of treatment, the geometric means of the number of parasitic elements before the first treatment and post-treatment, (i) 14 days for intestinal helminths, (ii) 28 and 56 days for lungworms and (iii) 28 days for *O. cynotis* and fleas, were estimated and compared. Following statistical analyses (paired *t*-test and McNemar’s test), an efficacy of 100% was recorded against the most commonly detected parasites (gastrointestinal helminths and mites) and a notable statistically significant effect against fleas and lungworms after one dose, while 100% efficacy against lungworms was achieved after two doses of the product. No adverse effects were reported. The prevalence of parasitism in owned cats in Greece remains high, highlighting the demand for targeted preventive antiparasitic schemes. This study demonstrated high-level efficacy and tolerance of NexGard^®^ Combo against common endoparasites and ectoparasites of household cats in Greece.

## Simple Summary

Pet cats can be infected with many internal and external parasites. These parasites may cause health problems in cats and some of them may also infect humans. In this study 472 household cats from Greece were examined for the presence of parasites. Furthermore, in positive animals an antiparasitic medication, namely NexGard^®^ Combo, was administered topically. This product combines three antiparasitic substances that kill internal and external parasites at the same time. Results confirmed that owned cats in Greece are often infected by parasites and that multiparasitism is common. This study also proved that NexGard^®^ Combo is efficient against the major parasites of cats in Greece and also safe and easy to use.

## 1. Introduction

Cats represent primary hosts for numerous metazoan parasites; both endoparasites such as nematodes (intestinal and respiratory) and cestodes and ectoparasites including fleas, mites and ticks [1,2]. Overall, feline parasitism affects the health and welfare status of animals, being a matter of prime concern for cat owners and veterinarians, since some of the parasites are widespread among client-owned cats across Europe [2,3]. This phenomenon can be attributed to the high pressure of parasites, particularly in animals spending time outdoors, and the use of narrow-spectrum antiparasitic drugs. Additionally, since some domestic cats may exhibit predatory behaviour, they are likely to acquire multiple parasites [2,4,5,6]. Indeed, a multicenter large-scale study (1519 cats) in Europe concluded that the prevalence of multiparasitism was up to 14% in privately owned cats [2]. Interestingly, several of these pathogens may have zoonotic implications and are important from a medical standpoint [7,8,9,10]. Several epizootiological surveys for feline parasitism have been conducted in Greece, studying various cats populations with different lifestyles [11,12,13,14,15,16,17].

The continuous monitoring of parasitism in cats and the update of its epizootiological traits are key points for the successful control of parasites, which includes antiparasitic treatments combined with targeted prevention strategies. Currently, research efforts against feline parasites have been focused on the proposal of efficient treatments without adverse reactions. As expected, veterinarians prefer easily administered, commercial products with a wide spectrum of antiparasitic activity. NexGard^®^ Combo (Boehringer Ingelheim) is a formulation designed to eliminate numerous feline endo- and ectoparasites, combining three compounds, namely esafoxolaner, eprinomectin and praziquantel. Esafoxolaner is the purified (S)-enantiomer of afoxolaner with acaricidal and insecticidal properties. Eprinomectin is an avermectin of the macrocyclic lactones class, efficient against the larval and adult stages of several nematodes, while praziquantel exhibits cestodicide properties [18]. To date, several studies have demonstrated the high efficacy of NexGard^®^ Combo against the main ecto- and endoparasites of cats but mainly in cases of mono-infections and less commonly in cases of multiparasitism [19,20,21,22,23,24,25,26,27,28,29,30,31,32,33,34]. In Greece there is a lack of data regarding the efficacy of this formulation against multiparasitism. Moreover, the safety of this product has been well documented in relevant studies [35,36].

The objectives of this study were: (i) to provide an update on the current epizootiology of the most common endo- and ectoparasites in client-owned cats in Greece and (ii) to confirm the efficacy and safety of NexGard^®^ Combo against local strains of the major endo- and ectoparasites in naturally parasitised cats in Greece.

## 2. Materials and Methods

### 2.1. Study Design

This study received approval by the Ethics Committee of the Aristotle University of Thessaloniki (326409/2023). Informed consent was obtained from the cat owners before the enrolment of the animals. Overall, 472 clinically healthy client-owned cats were examined between March 2023 and September 2024 during their routine visits to veterinary practices across Greece’s mainland to determine the epizootiology of feline parasitism. Data regarding living conditions, age, sex and breed were recorded for each animal. All cats were examined for the presence of endo- and ectoparasites once between Days -7 and 0 (mean Day -3) and, thereafter, all animals found to be positive for at least one parasite were included in the second part of the study, where treatment with NexGard^®^ Combo was applied (Day 0, which was considered as the baseline time). After treatment, cats were re-examined for the efficacy assessment (i) on Day 14 (±1) for intestinal helminths, i.e., *Toxocara cati*, hookworms and *Dipylidium caninum*, (ii) twice on Days 28 (±1) and 56 (±1) for lungworms and (iii) on Day 28 (±1) for *Otodectes cynotis* and fleas.

### 2.2. Examination for Endoparasites—Faecal Sample Collection and Coprological Methods Employed

None of the examined animals received anthelmintic treatment at least 3 months before their enrolment in the study. Faecal specimens were collected from each individual cat with the owner’s help, either under supervision at the time of elimination or fresh, directly from the animal’s litter box. All samples were placed in individual plastic containers, labelled, stored at 4 °C and examined within 48 h at the Laboratory of Parasitology and Parasitic Diseases of the School of Veterinary Medicine in Thessaloniki.

Each sample was macroscopically examined for the presence of cestode segments and/or adult nematodes. Thereafter, each specimen was subjected to a microscopic analysis using a standardised quantitative technique (a modified McMaster method), as described by Taylor et al. [37], with the following changes. In particular, the parasite burdens for ascarids, hookworms and cestodes were assessed by an egg-counting technique as follows: 3 g of faeces was mixed with 45 mL of distilled water (instead of 42 mL) until the fluid was homogeneous. Afterwards, the mixture was filtered through a sieve, and 15 mL of the filtrate was transferred to a tube. Following centrifugation at 800× *g* for 5 min (instead of 2 min), the supernatant was removed, and the sediment was resuspended in saturated NaCl (specific gravity 1.2) added to a final volume of 15 mL. Next, the suspension was collected with a pipette to fill both counting chambers of a McMaster slide. After 5 min, all parasitic elements inside the mesh areas of both chambers corresponding to a total volume of 0.3 mL of the suspension were counted under an optical microscope (Olympus CX21 Microscope, Evident Corporation, Tokyo, Japan) at 100× magnification and added up in groups. The total eggs per gram of faeces (EPG) for each category of parasites were calculated when each corresponding sum was multiplied by 50. Concerning *D. caninum,* in addition to the macroscopic observation for the presence of segments which is suggested as the most accurate diagnostic approach, egg counting was performed where each egg packet was considered to include a mean of 12 eggs [38] and calculations were made accordingly.

In addition, for all samples, a quantitative modified Baermann technique based on Thienpont et al. (1986) [39] was applied, where the number of L1 larvae per gram of faeces (LPG) was calculated as follows: 5 g of faeces was placed in a pouch created with double-layered gauze and submerged in a Baermann funnel filled with 45 mL of tap water. Faeces remained there for approximately one day for the L1 larvae to migrate. Then, for the recovery of the larvae, the solution was poured into a Falcon tube and centrifuged at 800× *g* for 5 min. Thereafter, the supernatant was discarded carefully by vacuum suction, and the whole sediment was microscopically examined. Larvae were morphologically identified under an optical microscope (at 100×, 400× and 1000× magnification when necessary), summed up and divided by 5 to assess the LPG.

All retrieved parasitic elements were identified based on morphological and morphometric features [37,40,41]. A cat was considered positive if at least one parasitic element (egg, larva or proglottid) was observed.

### 2.3. Examination for Ectoparasites

None of the animals had been treated with any ectoparasiticide (topical or systemic) within the last 3 months prior to Day 0. All handlings were performed in a calm and gentle manner to reduce stress during the visit. As regards infestation with *O. cynotis,* the presence or absence of mites was estimated by an otoscopic examination on each cat before the baseline time (Day -3), whereupon mites were counted to ensure that all eligible positive cats were sufficiently infested with a minimum of 3 live mites in at least one ear. Following treatment, the presence of viable stages of *O. cynotis* mites was assessed on Day 28, and mites were counted; for this purpose, sedation and flushing of the ear canals using mineral oil was performed and the aural canal debris along with exudates were microscopically examined [42].

The presence of viable *Notoedres cati* mites was confirmed after collecting deep skin scrapings using a scalpel blade from three areas of at least 1 cm × 1 cm at body sites suspected of being infested. Samples were microscopically examined within 24 h, and larvae, nymphs and adult *N. cati* mites were identified based on morphological characteristics [40].

The presence of fleas was evaluated by brushing the coat of cats for a minimum of 5 min from head to tail, while, at the same time, attention was paid to areas of the body most likely to harbour these arthropods, such as the neck, ventral face, lower back and inner thighs. The fine-tooth flea comb was dampened to increase the chance of fleas being trapped. After grooming, all gathered material from each cat was put in a plastic bag and transferred to the laboratory. The examination was performed by visualisation of live fleas. The presence of fleas and the species were determined under a stereomicroscope (Olympus, Research Stereomicroscope System SZH10, Hamburg, Germany) (8×–64×) and a light microscope (Olympus CX21 Microscope, Evident Corporation, Tokyo, Japan) (100× and 400×) using established morphological identification criteria [43]. Flea counts were performed for all positive cats on Day -3 and repeated on Day 28.

All cats were examined for the presence of ticks by thorough inspection all over the cat’s body to detect any small lumps. Attention was paid to usual attachment areas with thinner skin, such as around and inside the ears, under the collar, between the toes, under the front legs, behind the back legs and around the tail and rectum. Collected ticks were identified to the genus and species level using a stereomicroscope (8×–64×) based on morphological traits and in line with standardised taxonomic keys [40].

### 2.4. Treatment Administration

All cats harbouring at least one parasitic element were considered eligible for the study and treated with NexGard^®^ Combo topical solution once on Day 0. In particular, all infected/infested cats were treated once on Day 0, except for animals infected with lungworms, which received treatment twice during the course of the study on Days 0 and 28.

Cats were weighed, and the spot-on treatment was applied according to the recommendations of the product, at the dosages of 0.3 mL for animals weighing 0.8 to <2.5 kg and 0.9 mL for animals weighing 2.5 to <7.5 kg. At these dosages, NexGard^®^ Combo delivers 3.60/10.80 mg esafoxolaner, 1.20/3.60 mg eprinomectin and 24.90/74.70 mg praziquantel. The application site was on the neck skin, after parting the hair, in a spot located on the midline between the skull base and the shoulder blades.

### 2.5. Acceptability of Treatment—Safety Evaluation

All eligible cats were physically examined at enrolment (before treatment), at each scheduled visit (Day 14 for intestinal helminths, Days 28 and 56 for lungworms and Day 28 for *O. cynotis* and fleas) and at the end of the study. Furthermore, the cat owners observed their pets closely for the first 3 h post-treatment and once a day until the completion of the study to record potential side-effects likely to be associated with the treatment. At study completion, the acceptability of treatment based on the physical examinations of the animals and the occurrence of any reported adverse effects were assessed.

### 2.6. Data Handling: Statistical Analyses

Data were collected in Microsoft Excel spreadsheets, appropriately designed for the subsequent analyses (Additional File S1). For the estimation of the prevalence values and their 95% confidence intervals (95% CIs), the Epitools (ausvet.com.au) and the Wilson score interval methods were used. For the evaluation of the treatment efficacy, a paired *t*-test and McNemar’s test were used on quantitative (EPG for intestinal helminths, LPG for lungworms and number of mites, pre- and post-treatment) and qualitative data, respectively, in SPSS v23. Statistical significance was set at the α = 0.05 level.

## 3. Results

### 3.1. Sampled Cat Population

In total, 472 cats were examined for the presence of parasitic elements (244 (51.7%) females and 228 (48.3%) males). Concerning age, 171 cats (36.2%) were <1 year old, 151 (32.0%) were between 1 and 5 years old and 150 (31.8%) were >5 years old. The majority of animals (n = 459 cats; 97.2%) were Domestic Shorthairs (DSHs) while seven (1.5%), four (0.9%) and two (0.4%) of them belonged to the breeds Siam, Maine Coon and Persian, respectively. Regarding lifestyle, 34.3% (n = 162 cats) of the studied population lived outdoors, 36.0% (n = 170 cats) lived inside and were allowed to free-roam and 29.7% (n = 140 cats) were kept strictly indoors.

### 3.2. Prevalence of Endoparasite Infections and Ectoparasite Infestations in the Cats Examined for Enrolment

The overall prevalence of parasitism (single or mixed infections/infestations) in the studied cat population was 22.9% (108/472); in particular, 6.6% (31/472) and 16.3% (77/472) for single and mixed infections/infestations, respectively. The infection rate with endoparasites was 22.2% (105/472) and the infestation rate with ectoparasites was 6.6% (31/472). In infected cats, the ranges (and mean ± standard deviation values) of *T. cati*, hookworms, *D. caninum* EPG and lungworm LPG were 50–600 (222 ± 138.0), 50–800 (337 ± 182.2), 150–600 (332 ± 129.6) and 16–62 (39 ± 16.1), respectively (Additional File S1). In the studied cat population, 15 cats were found infested by *O. cynotis* mites (>5 mites) and 11 by fleas (range: 6–22; mean ± standard deviation: 13.0 ± 5.4). Prevalence values of parasitism according to the lifestyle, age, sex and overall are summarised in Table 1.

The most frequently identified endoparasites were the nematodes *T. cati* (n = 87; 18.4%), hookworms (n = 51; 10.8%) and lungworms (n = 12; 2.5%) and the cestode *D. caninum* (n = 22; 4.7%). In addition, infection with the ascarid *Toxascaris leonina* was identified in two cats (0.4%). The most frequently identified ectoparasites were *O. cynotis* (n = 15; 3.2%) and fleas (n = 11; 2.3%). Also, infestations with the mite *N. cati* and ticks were recorded in two (0.4%) and three (0.6%) animals, respectively (Table 2).

### 3.3. Evaluation of Efficacy

From the initially enrolled cat population, overall, 108 cats were eligible for treatment with NexGard^®^ Combo. In most cases, a single treatment against endo- and ectoparasites was 100% efficient (no parasitic elements were found post-treatment), except for lungworms and fleas, where *t*-values of 8.61 (df = 11, *p* < 0.001) and 7.72 (df = 10, *p* < 0.001), respectively, were estimated, still indicating a high efficiency after the first treatment; this was also confirmed by the McNemar’s test results which revealed a significant difference in improvement rates before and after the treatment for lungworms and fleas (*p* < 0.01 in both cases) after the first treatment. The second treatment applied against lungworms, 28 days after the first treatment, was 100% efficient. Finally, although the effectiveness of treatment for rare parasites like *N. cati* was evidenced herein, additional large-scale confirmatory studies are required to address remaining uncertainties.

### 3.4. Evaluation of Safety

All cats completed the study. The safety of the product was proved according to the physical examinations performed by the veterinary practitioners and observation performed by cat owners. Neither health abnormalities nor clinical manifestations were witnessed post-treatment, which might have been treatment-related, either by the owners or the vets.

## 4. Discussion

Domestic cats may be infected with a wide range of clinically important endo- and ectoparasites and feline parasitism represents a topic of increasing interest for veterinarians. The present study provides an updated view of the epizootiology of the major feline parasites in Greece. Overall, the prevalence of parasitism was 22.9%. This is lower compared to the prevalence rate estimated by data collected during a survey conducted across Europe, which demonstrated that more than half of pet cats (50.7%) harboured at least one parasite at a given time point [2]. The unexpectedly increased prevalence rate is an impressive fact considering that these animals are mostly living indoors and are expected to be well taken care of by their owners in terms of the applied preventive veterinary measures. Our study showcases the multispecies nature of feline parasitism in our country with 16.3% of cats demonstrating multiparasitism. This is consistent with the findings reported by the aforementioned epizootiological survey in Europe which highlighted for the first time that multiparasitism is common in client-owned cats, demonstrating that 14.0% of the cats were co-infected with endo- and ectoparasites, with 11.9% of them harbouring both gastrointestinal helminths and ectoparasites [2]. Additionally, it is important to underscore the zoonotic considerations of several feline parasites that render their effective control and treatment of pivotal significance not only for animals but also for mitigating the risk for public health [7,8,9,10,44]. Considering the above, one realises the importance of effective treatment for both endo- and ectoparasites of cats using a product that combines such treatment claims [6].

NexGard^®^ Combo is an endectoparasiticide the European Medicine Agency licensed on 13 January 2021. This treatment option contains the active compounds esafoxolaner, eprinomectin and praziquantel [18], and it has been proven to offer a broad spectrum of antiparasitic activity in previous efficacy surveys [19,20,21,22,27,28,29,30,31,32,33,34]. The present study extends related research by confirming the broad endectoparasiticide effectiveness of the product and its acceptability by simultaneously assessing its nematodicide, cestodicide and ectoparasiticide properties in Greek-owned naturally infected/infested cats.

### 4.1. Gastrointestinal Helminths (Nematodes and Cestodes)

Among parasites, gastrointestinal helminths are frequently found in cats [9,45,46]. In Europe, the probability of client-owned cats being infected by any type of gastrointestinal helminth appears to be higher than expected; indeed, several European studies have revealed concurrent nematode and cestode infections in between 5% and 14% of household cats [5,6,47], while even higher prevalence values have been reported in stray cats [2]. Due to the modalities of infection (e.g., ingestion of eggs or larvae, hunting small animals), household cats that usually go outdoors are obviously at greater risk of exposure to both nematode and cestode infections [2,4,5,6]. Interestingly, it has been evidenced that even cats without outdoor access are at risk of helminth infections. In a cross-sectional study conducted in Germany and France, 20% of pet cats that were positive for helminth infection did not have outdoor access [48].

The most common digestive helminth encountered in cats worldwide is *T. cati*, with a 17.0% global pooled prevalence when copromicroscopy is employed [49]. Indeed, European studies observed that *T. cati* was the most common parasite in privately owned cats, with its prevalence ranging from 2.9 to 19.7%. In particular, in Greece, a systematic literature review and meta-analysis reported a mean prevalence of 18.2% [49] for *T. cati* infection and a more recent one a similar rate of 17.3% [50]. However, it should be noted that research on free-roaming and household Greek cats was included in both of these meta-analysis studies. Concerning client-owned Greek cats, a study examining 205 owned animals demonstrated 7.8% *T. cati* infection [14]; this percentage rose significantly to 20.7% when 560 cats were examined [11]. In agreement with the aforementioned large-scale studies, our study concluded that 18.4% of the owned cats were infected. Our findings, coupled with the results by retrospective study by Nijsse et al., which concluded that even if cats spend less than an hour per day outdoors they are at greater risk for infection acquisition [51], point out the roaming of cats as a major risk factor.

Regarding the pathogenicity of *T. cati* infections, adult animals do not present clinical signs [38]. In kittens or in cases of massive infections, *T. cati* may induce clinical disease with listlessness, cachexia, diarrhoea, vomiting, reduced growth rate, anaemia, cough due to larval pulmonary migration and sometimes even death [9,45,52]. Noteworthily, in owned cats with digestive clinical signs, *T. cati* was the most frequently identified parasite, with a striking prevalence of 40.2% [53]. Besides its veterinary importance, this nematode has zoonotic potential [8,54].

The other ascarid species of cats, namely *T. leonina,* is rarely reported in client-owned cats, which is consistent with the results of our study (0.4%) [6]. Except for Diakou et al. (2017) [13] reporting an 8.0% prevalence of *T. leonina* in stray cats, the rest of the studies either failed to detect the parasite [14] or detected it with a minimal infection rate, i.e., 0.1% [11] in Greek cats.

Cats may become infected with different hookworm species, including *Ancylostoma tubaeforme*, the main species identified, and *Ancylostoma ceylanicum* or *Ancylostoma braziliense*, found in tropical countries. These nematodes usually establish subclinical infections or mild digestive disorders in cats; nonetheless, they may impair the growth and welfare of the animal. When heavy parasitism with these blood-sucking parasites occurs, haemorrhagic enteritis, hypoalbuminaemia and anaemia may manifest [55]. The zoonotic implications of hookworms are well documented and highlight their importance for public health [56,57].

*Ancylostoma tubaeforme* is enzootic in cats throughout the world. In Europe, the prevalence ranges from 1% up to 44.4% depending on the examined cat population [5,6,58,59]. In our study, almost one out of ten (10.8%) cats was found infected with hookworms, which is similar to the infection rate (8.3%) reported by Kostopoulou et al. in Greece a few years ago [14]. However, when many specimens are examined, the infection rate is even lower, as depicted in a cross-sectional large-scale study (4.2%) [11].

One of the major cestodes of cats is *D. caninum*. In a European study PCR testing was employed to detect the presence of *D. caninum* in fleas from client-owned cats, unveiling that infections of cats were likely to occur in households [60]. However, *D. caninum* infections scarcely cause severe clinical manifestations in cats unless there is a high parasitic burden [40]. Despite the diagnostic limitations of *D. caninum* infections, the recorded prevalence in the current study was 4.7%.

NexGard^®^ Combo contains two anthelminthic substances of well-documented safety and efficacy against a broad spectrum of feline gastrointestinal helminths [61,62,63,64,65], i.e., eprinomectin and praziquantel. Indeed, following a topical application of the product, the eprinomectin and praziquantel plasma profiles demonstrate adequate systemic endoparasiticide concentrations required to eliminate nematodes and cestodes, sustaining NexGard^®^ Combo activity without being impacted by the isoxazoline compound esafoxolaner [66].

In our study, a single dose of NexGard^®^ Combo prevented egg shedding, providing 100% efficacy, as assessed by the quantitative McMaster technique. Consistent with our results, several studies using experimentally or naturally infected cats in Europe, South Africa and North America have documented that a single administration of NexGard^®^ Combo at the minimum label dosage eliminated >98% and >93% of adult *T. cati* and *D. caninum*, respectively [23]. Our study showcases that eprinomectin maintains its excellent systemic activity as well as its bioavailability when applied topically, confirming the results of relevant pharmacokinetics studies [67].

### 4.2. Lungworms

Feline lungworms are among the cat parasites that have gained interest in the last decade. The most commonly studied ones are the metastrongyloids *Aelurostrongylus abstrusus* and *Troglostrongylus brevior* which challenge the health and welfare of cats [41,68]. In aelurostrongylosis, a variety of clinical features ranging from mild to evident ones, including sneezing, wheezing, coughing, tachypnea and dyspnoea, may be present, while in severe cases conditions that threaten the life of the animal have been described [69,70]. *Troglostrongylus brevior* has limited clinical relevance in adult animals, while it is pathogenic in kittens [68], with fatal outcomes being observed in severe cases [71,72,73].

With reference to the prevalence of feline lungworms in privately owned cats in 2017, a metacentric European study examined 1990 animals and concluded that at least one out of ten (10.6%) was infected [3]. In our study, 2.5% of household cats were exposed to metastrongylids, which is very close to the prevalence reported in a large-scale survey (3.2%) regarding owned cats [11]. Preceding surveys have already confirmed the presence of these parasites in Greek cats, with a higher infection rate (12.0%) being found in stray animals [15].

Previous studies have demonstrated high safety and efficacy of eprinomectin in interrupting lungworm larval shedding in cats under natural conditions in both single and mixed infections [74,75,76]. This was confirmed in the present study, where complete elimination of lungworm larvae was observed after two administrations of NexGard^®^ Combo in all animals. These results are in agreement with a study confirming that two doses of NexGard^®^ Combo at 28 days apart interrupted L1 shedding in all the studied cats [27]. Notably, NexGard^®^ Combo has additionally been proven highly effective in terms of clinical recovery and resolution of radiographic findings of cats with natural infections, since a study showed that almost all infected animals which received this spot-on formulation completely recovered from clinical disease and radiographic alterations within 2 months after treatment [27]. One could speculate that efficacy evaluations based solely on reducing larval shedding in faeces may be inaccurate since parasiticides may temporarily disturb parasite fertility. However, several studies have demonstrated that most of the experimentally infected cats, which did not shed lungworm larvae after treatment, were also negative for adult parasites or had dead worms at necropsy [74,75,77,78]. Therefore, the copromicroscopic examinations in this research robustly support the successful disappearance of adult parasites in the studied cats with natural infections.

### 4.3. Mites Otodectes cynotis and Notoedres cati

*Otodectes cynotis* (family Psoroptidae) mites represent the leading cause of otitis externa [79], and so the typical clinical picture of otoacariosis includes amounts of brown discharge inside the ear canal with varying degrees of pruritus and erythema [40]. It has been reported that *O. cynotis* accounts for up to 85% of otitis externa cases in cats [43].

In a multicentric and large-scale survey performed in 1519 owned cats in Europe, ear mites were the most frequently identified ectoparasites (17.4%) [2]. Accordingly, infestations by *O. cynotis* have been recorded in the past in client-owned kittens and young cats in Greece at a prevalence of 14.0% [16], which is higher compared to our findings. Another survey in Greece recorded a 13.6% infestation rate in cats living indoors without contact with other pets and a much higher 42% in the case of indoor cats that shared a household with other pets [17].

Another mange mite of clinical relevance in cats is *N. cati* (family Sarcoptidae). Notoedric mange manifests firstly with signs such as pruritus, papules, alopecia and hyperkeratosis of the outer ear that may rapidly extend to the face, neck, feet and perineum of the animal. If left untreated, secondary bacterial or yeast infections develop at the site of the self-trauma lesions due to pruritus [40,80]. *Notoedres cati* has also been reported to infest non-felid hosts, including humans [81]. Actual prevalence data are scarce in Greece, and there are no studies regarding pet cats, except for this study, where the infestation rate was relatively low (0.4%).

Successful management of otodectic or notoedric mange in cats requires effective acaricides. New curative solutions against mites arose with the compounds of the isoxazoline class such as afoxolaner, fluralaner and sarolaner. Off-label use of oral afoxolaner has also been proven effective in treating otodectic mange in cats [82]. NexGard^®^ Combo contains esafoxolaner, an active (S)-enantiomer of afoxolaner, and a single treatment with this formulation has been proven to be over 97% effective against *O. cynotis* in both natural and induced infestations in two laboratory trials and in one field study [31]. The therapeutic efficacy of NexGard^®^ Combo was also demonstrated against otodectic mange in a controlled clinical study, where one treatment eliminated all live mites, and there was a complete clinical recovery within 42 days [31]. In the same frame, the efficacy of a single administration of this formula was proven in treating notoedric mange in cats under in-home conditions [36]. The present study verifies the above, since a single dose of NexGard^®^ Combo killed all *O. cynotis* mites within 28 days after treatment, thus providing robust evidence of the efficacy of this formulation against otodectic mange.

### 4.4. Blood-Sucking Ectoparasites—Fleas and Ticks

Flea infestations constitute a significant veterinary and public health concern. *Ctenocephalides felis* is the main flea species identified in cats globally [10]. Animals infested by fleas may exhibit discomfort, pruritus, self-wound formation, blood loss and subsequent anaemia [38]. Except for its clinical impact, *C. felis* is the vector of pathogens, some of which have zoonotic potential, i.e., *Bartonella henselae*, the agent of cat scratch disease, *Rickettsia felis*, the agent of spotted fever, and *Yersinia pestis*, the agent of plague [38]. Regarding flea prevalence in owned cats, a European cross-sectional survey including a high number of animals demonstrated that 15.5% of them were infested [2]. Given the particularities of flea detection in live cats, data are scarce in our country. In the present study, the prevalence of flea-infested animals was 2.3% and as expected was lower compared to the prevalence in stray cats reported in a past survey (24.3%) [12]. In both aforementioned studies, the only species identified was *C. felis*, which is in accordance with another study in Greece where almost all of the studied infested animals (97.4%) harboured *C. felis* [83].

Today, a broad range of active molecules and commercial products are available to control flea populations. Afoxolaner binds on the insect *γ*-aminobutyric acid (GABA) receptor and glutamate receptors, thus inhibiting ligand-gated uptake of chloride ions, thereby inducing excess neuronal stimulation and subsequent death of the arthropod [84]. This molecule has been proven efficacious against adult fleas and flea egg production in infested dogs [85,86,87]. The insecticidal efficacy of NexGard^®^ Combo has been illustrated for adult fleas in experimental [30] and field studies [4,24]. In line with the above surveys, in our study, all animals scored negative for fleas 28 days post-treatment following a single administration of NexGard^®^ Combo. Interestingly, in two experimental studies, NexGard^®^ Combo was also demonstrated to be efficacious against the inhibition of flea larval development and egg production [30], which is substantive since this formulation breaks the lifecycle of fleas at multiple stages [88]. Another important feature of NexGard^®^ Combo is that it provides immediate flea-killing activity. This quick activity is pivotal for the efficacy of a drug against fleas since the speed of kill has been correlated with the risk of transmitting vector-borne agents [89].

In the present study, only 0.6% of the household cats harboured ticks, particularly *Rhipicephalus sanguineus*. The corresponding prevalence for stray cats in Greece was 0.88% [12]. The pathogenicity of ticks is associated with their blood-feeding behaviour. Along with inflammatory reactions at the bite site, heavy infestations may result in anaemia in infested animals. Overall, ticks are of concern as vectors of agents affecting both cats and humans [38]. The efficacy of NexGard^®^ Combo against these ticks has been established in preceding studies [21,34].

To summarise, feline parasitism, single or multiparasitism, remains common in client-owned Greek cats. Therefore, attention must be drawn from veterinarians to raise awareness and adequately educate cat owners. Nevertheless, such studies including a big number of animals might have potential limitations. For example, we examined only one sample per animal before inclusion and not pooled samples of consecutive days and so the risk of underdetection cannot be excluded. This field trial confirms that NexGard^®^ Combo offers a broad endectoparasiticide spectrum against feline parasites in Greece, and therefore, it is recommended as a reliable treatment option in clinical veterinary practice. The control of multiple concurrent parasitic infections and/or infestations is important for the improvement of the health and welfare of cats and the protection of their owners. Moreover, no adverse reactions related to NexGard^®^ Combo administration were observed, thus corroborating previous reports on the high-level tolerance of this formulation.

## 5. Conclusions

The present study confirms that parasitism in client-owned cats in Greece remains common despite the animal’s lifestyle. This field trial demonstrated that a formulation combining esafoxolaner, eprinomectin and praziquantel (NexGard^®^ Combo) is highly effective against major metazoan parasites of cats in Greece. These results are consistent with preceding surveys and unwaveringly support the idea that this treatment option provides veterinarians and cat owners with an effective tool for controlling single or multiple parasitoses in cats. Furthermore, this therapeutic approach displayed an excellent safety profile and was convenient to administer, all features that help to enhance owner compliance.

## Figures and Tables

**Table 1:** Prevalence values (means and 95% confidence intervals) of parasitic infections/infestations according to lifestyle, age, sex and overall.

	Factor	Factor Group	Number of Positive Animals	Number of Tested Animals	Prevalence (%)	95% CI of Prevalence (%)
Parasitic infection/infestation	Lifestyle	Outdoors	58	162	35.8	28.8–43.4
		Outdoors/Indoors	34	170	20.0	14.7–26.7
		Indoors	16	140	11.4	7.2–17.8
	Age	<1 year	41	171	24.0	18.2–30.1
		1–5 years	31	151	20.5	14.9–27.7
		>5 years	36	150	24.0	17.9–31.4
	Sex	Female	52	244	21.3	16.6–26.9
		Male	56	228	24.6	19.4–30.5
	Overall prevalence	-	108	472	22.9	19.3–26.9
Single infection/infestation	Lifestyle	Outdoors	16	162	9.9	6.2–15.4
		Outdoors/Indoors	10	170	5.9	3.2–10.5
		Indoors	5	140	3.6	1.5–8.1
	Age	<1 year	9	171	5.3	2.8–9.7
		1–5 years	8	151	5.3	2.7–10.1
		>5 years	14	150	9.3	5.6–15.1
	Sex	Female	16	244	6.6	4.1–10.4
		Male	15	228	6.6	4.0–10.6
	Overall prevalence	-	31	472	6.6	4.7–9.2
Mixed infection/infestation	Lifestyle	Outdoors	42	162	25.9	19.8–33.2
		Outdoors/Indoors	24	170	14.1	9.7–20.2
		Indoors	11	140	7.9	4.4–13.5
	Age	<1 year	32	171	18.7	13.6–25.2
		1–5 years	23	151	15.2	10.4–21.8
		>5 years	22	150	14.7	9.9–21.2
	Sex	Female	36	244	14.8	10.9–19.8
		Male	41	228	18.0	13.5–23.5
	Overall prevalence	-	77	472	16.3	13.3–19.9

CI: Confidence Interval.

**Table 2:** Prevalence values (means and 95% confidence intervals) of parasitic infections/infestations counting >10 cases in the studied cat population (*Toxocara cati*, hookworms, lungworms, *Dipylidium caninum*, *Otodectes cynotis* and fleas) per parasite and according to the lifestyle, age, sex and overall.

	Factor	Factor Group	Number of Positive Animals	Number of Tested Animals	Prevalence (%)	95% CI of Prevalence (%)
*Toxocara cati*	Lifestyle	Outdoors	46	162	28.4	22.0–35.8
		Outdoors/Indoors	29	170	17.1	12.2–23.4
		Indoors	12	140	8.6	5.0–14.4
	Age	<1 year	33	171	19.3	14.1–25.9
		1–5 years	25	151	16.6	11.5–23.3
		>5 years	29	150	19.3	13.8–26.4
	Sex	Female	40	244	16.4	12.3–21.6
		Male	47	228	20.6	15.9–26.3
	Overall prevalence	-	87	472	18.4	15.2–22.2
Hookworms	Lifestyle	Outdoors	30	162	18.5	13.3–25.2
		Outdoors/Indoors	14	170	8.2	5.0–13.4
		Indoors	7	140	5.0	2.4–10.0
	Age	<1 year	20	171	11.7	7.7–17.4
		1–5 years	16	151	10.6	6.6–16.5
		>5 years	15	150	10.0	6.2–15.8
	Sex	Female	23	244	9.4	6.4–13.8
		Male	28	228	12.3	8.6–17.2
	Overall prevalence	-	51	472	10.8	8.3–13.9
Lungworms	Lifestyle	Outdoors	10	162	6.2	3.4–11.0
		Outdoors/Indoors	2	170	1.2	0.3–4.2
		Indoors	0	140	0.0	0.0–2.7
	Age	<1 year	7	171	4.1	2.0–8.2
		1–5 years	2	151	1.3	0.4–4.7
		>5 years	3	150	2.0	0.7–5.7
	Sex	Female	5	244	2.0	0.9–4.7
		Male	7	228	3.0	1.5–6.2
	Overall prevalence	-	12	472	2.5	1.5–4.4
*Dipylidium caninum*	Lifestyle	Outdoors	10	162	6.2	3.4–11.0
		Outdoors/Indoors	8	170	4.7	2.4–9.0
		Indoors	4	140	2.9	1.1–7.1
	Age	<1 year	7	171	4.1	2.0–8.2
		1–5 years	7	151	4.6	2.3–9.3
		>5 years	8	150	5.3	2.7–10.2
	Sex	Female	14	244	5.7	3.4–9.4
		Male	8	228	3.5	1.8–6.8
	Overall prevalence	-	22	472	4.7	3.1–7.0
*Otodectes cynotis*	Lifestyle	Outdoors	4	162	2.5	1.0–6.2
		Outdoors/Indoors	10	170	5.9	3.2–10.5
		Indoors	1	140	0.7	0.1–3.9
	Age	<1 year	5	171	2.9	1.3–6.7
		1–5 years	5	151	3.3	1.4–7.5
		>5 years	5	150	3.3	1.4–7.6
	Sex	Female	10	244	4.1	2.2–7.4
		Male	5	228	2.2	0.9–5.0
	Overall prevalence	-	15	472	3.2	1.9–5.2
Fleas	Lifestyle	Outdoors	2	162	1.2	0.3–4.4
		Outdoors/Indoors	2	170	1.2	0.3–4.2
		Indoors	7	140	5.0	2.4–10.0
	Age	<1 year	3	171	1.8	0.6–5.0
		1–5 years	3	151	3.0	0.7–5.7
		>5 years	5	150	3.3	1.4–7.6
	Sex	Female	3	244	1.2	0.4–3.6
		Male	8	228	3.5	1.8–6.8
	Overall prevalence	-	11	472	2.3	1.3–4.1

CI: Confidence Interval.

## Data Availability

The original contributions presented in the study are included in the article. Further inquiries can be directed to the corresponding author.

## Supplementary Materials

The following supporting information can be downloaded at: https://www.mdpi.com/article/10.3390/vetsci12040385/s1, Additional File S1: Excel datasheet.
